# Influence of Crosslink Density on Electrical Performance and Rheological Properties of Crosslinked Polyethylene

**DOI:** 10.3390/polym16050676

**Published:** 2024-03-01

**Authors:** Linting Di, Chenyuan Qin, Wenying Wang, Anping Huang, Fuqing Wei, Huifang Xu, Shiyuan Yang

**Affiliations:** Lanzhou Petrochemical Research Center, Petrochemical Research Institute, PetroChina, Lanzhou 730060, China; qinchenyuan12@petrochina.com.cn (C.Q.); wangwenying1@petrochina.com.cn (W.W.); huanganping@petrochina.com.cn (A.H.); weifuqing@petrochina.com.cn (F.W.); xuhuifang@petrochina.com.cn (H.X.); yangshiyuan@petrochina.com.cn (S.Y.)

**Keywords:** cross-linked structure, rheological, branch, crystal, liquation

## Abstract

To investigate the influence of the crosslinked polyethylene (XLPE) structure on electrical performance, various analytical methods were employed to study polyethylene structures with different degrees of crosslinking. Dynamic rheological analysis was conducted to determine material shear viscosity, dynamic viscosity, storage modulus (G′), loss modulus (G″), and other rheological parameters. Additionally, the electrical performance of the material was analyzed by studying the phenomenon of space charge accumulation under direct current voltage. The results indicate that with an increasing mass fraction of the crosslinking agent, the crosslink density of crosslinked polyethylene initially increases and then decreases. When the dicumyl peroxide (DCP) content exceeds 1.0 wt.%, there is an accumulation of like-polarity space charges. The best rheological processing performance of crosslinked polyethylene is observed when the DCP content is in the range of 1.0–1.5 wt.%.

## 1. Introduction

Crosslinked polyethylene (XLPE) finds widespread applications in the field of wires and cables, with continuous improvements and developments in crosslinking processes and materials [1,2]. The materials are now easier to process and use, exhibiting more reliable performance, and are conducive to transportation and storage. Various crosslinking methods [3,4,5,6,7], including peroxy chemical crosslinking, silane crosslinking, irradiation crosslinking, and ultraviolet light crosslinking, have been employed.

In theory, any compound capable of inducing the generation of free radicals on the polyethylene macromolecular chain can serve as a free radical initiator. To enhance the processing window of uncrosslinked polyethylene cable materials and prevent premature crosslinking and burning, crosslinking agents used in cable production need to have a high decomposition temperature, with a half-life temperature requirement around 100 °C. In domestic applications, di-cumyl peroxide (DCP) is a commonly used crosslinking agent in the production of crosslinked polyethylene cable insulation materials. To ensure product quality [8,9,10], it is essential to judiciously choose the amount of crosslinking agent, minimizing it while meeting performance requirements to reduce the content of crosslinking by-products and ensure pressure resistance levels. The crosslinking agent in polyethylene decomposes into highly reactive free radicals, initiating a series of free radical reactions that result in the formation of XLPE [11,12]. Due to its three-dimensional network structure, XLPE is thermosetting material, exhibiting excellent heat resistance with a long-term operating temperature reaching 95~100 °C. The formation of chemical crosslinks between XLPE molecular chains enhances its mechanical properties, particularly hardness, stiffness, and wear resistance, compared to polyethylene [13,14].

This research introduces a novel approach to the selection of crosslinking agents and additives influencing the structure of polyethylene, aiming to enhance the processing window of uncrosslinked polyethylene cable materials, thereby preventing premature crosslinking and combustion. This study provides valuable insights for the future design and preparation of wire and cable materials. Crosslinking of low-density polyethylene was conducted using varying concentrations of di-cumyl peroxide (DCP). Subsequently, a series of material characterization techniques were employed to analyze aspects such as composition, microstructure, and macroscopic properties. Rheological and space charge characteristics of differently structured crosslinked polyethylene were compared. The experimental results were utilized to analyze the impact of the structure on the electrical properties of crosslinked polyethylene. The innovative approach in the selection of crosslinking agents and additives in this study, along with the comprehensive analysis of the effects on polyethylene structure and electrical properties, contributes valuable knowledge to the field of wire and cable materials. This investigation aims to guide future designs and preparations of materials in this domain.

## 2. Experimental Details

### 2.1. Sample Preparation

A total of 100 g of low-density polyethylene (LDPE) (Lanzhou Petrochemical, Lanzhou, China), antioxidant 300 (Hubei Xin Dividend Chemical, Hubei, China), 2,4-diphenyl-4-methyl-1-pentene (AMSD) (Lanzhou auxiliary factory, Lanzhou, China), and 0.5 g, 1.0 g, 1.5 g, and 2.0 g of dicumyl peroxide (DCP) (Aladdin, Shanghai, China) were weighed, corresponding to DCP mass fractions of 0.5%, 1.0%, 1.5%, and 2.0% wt.%, Table 1. Respectively, processing and extrusion were carried out using an extruder. The pellets were heated at 180 °C for 30 s in a small hot press and then hot-pressed at 180 °C for 10 min.

### 2.2. Crosslinking Degree Measurement

Samples with masses ranging from 0.5 g to 1.0 g were weighed (recorded as m_2_) and placed in a sieve (the sieve weight recorded as m_1_). The sieve, containing the sample, was suspended in a round-bottom flask using a metal wire, ensuring the sample was completely immersed in xylene. A reflux condenser was installed, and the heating device was turned on to heat the solvent to its boiling point while refluxing for 8 h. The metal wire with the sieve bag was carefully removed and placed in an oven at 140 °C for 3 h. After cooling the sieve bag and metal wire to room temperature, the mass of the sieve bag was measured and recorded as m_3_. The crosslinking degree was calculated using the formula shown in Equation (1).

(1) $$\text{crosslinking degree}=\frac{m_{3}-m_{1}}{m_{2}-m_{1}}\times 100\%$$

### 2.3. Branching Degree Measurement

High-temperature liquid nuclear magnetic resonance (NMR) tests were conducted on samples 3 and 4 using a Bruker Avance III 500 MHz spectrometer (Bruker, Karlsruhe, Germany).

Test conditions: solvent—deuterated ortho-dichlorobenzene; temperature—120 °C; scans—512 scans were accumulated (approximately 1 h) to obtain the ^13^C spectrum of the sample.

The grafting density represents the number of branches per 1000 carbons on the main chain, indicating the branching degree. The calculation method is illustrated in Equation (2).

(2) $$\text{branching degree}=\frac{I_{br}}{I_{br}+I_{\left(CH_{2}\right)}}\times 100\%$$

### 2.4. Melt Crystallization Analysis

A small amount of the sample was placed on a glass slide, melted on a hot stage at 230 °C, covered with another glass slide, and pressed into a thin film. After maintaining a constant temperature for 5 min to eliminate thermal history, the hot stage temperature was rapidly lowered to 90 °C. The crystalline morphology was observed under a polarizing microscope (OLYMPUS GX71) (Evident, Tokyo, Japan).

### 2.5. Rheological Behavior Study

Dynamic rheological tests were performed on XLPE-1 and 2 using a rotational rheometer (Haake Mars60) (ThermoFisher Scientific, Waltham, MA, USA).

Test conditions: strain sweep—stress γ value was confirmed at a test temperature of 130 °C to ensure subsequent tests were within the linear viscoelastic region.

Temperature sweep—conducted between 130–180 °C with a testing frequency ω = 10 rad/s. Based on the previously determined strain γ values, the relationship between the material’s storage modulus G′ and temperature was obtained at a heating rate of 2 °C/min.

Time sweep—dynamic time sweeps were conducted at temperatures of 140 °C and 160 °C. The evolution of dynamic rheological parameters over time was examined at a frequency of ω = 0.5 rad/s for a duration of 0–10,000 s.

### 2.6. Thermal Analysis

The thermal performance and crystallinity of crosslinked samples were investigated using a differential scanning calorimeter (DSC).

Instrument model: Mettler DSC3.

Test conditions: conducted under high-purity nitrogen protection. The sample was isothermally maintained at 30 °C for 5 min to eliminate thermal history. Then, it was heated at a rate of 10 °C/min to 200 °C, held for 2 min, and subsequently cooled at a rate of 5 °C/min to 30 °C. Both the heating and cooling curves were recorded.

Crystallinity was determined using the melting enthalpy method (peak area of the melting peak) with the formula shown in Equation (3). The value of Δ*H_m_* for 100% crystalline polyethylene was taken as Δ*H_m_* = 293 J∙g^−1^.

(3) $$\chi_{c}=\frac{∆H}{{∆H}_{m}}\times 100\%$$

### 2.7. Crystalline Performance Analysis

Instrument: Rigaku MiniFlex600 (manufactured in Rigaku, Tokyo, Japan).

Test conditions: scanning was performed in the range of 2θ = 0° to 100° at a rate of 0.05°/min using CuK α radiation.

For crystallinity calculation, a common method is the “Segal method” (also known as the Segal formula). This method estimates the ratio of crystalline to non-crystalline regions by measuring peak intensities.

The calculation formula for the Segal method is given in Equation (4):

(4) $$\text{Crystallinity}\left(\%\right)=\frac{I_{crys}}{I_{crys}+I_{amorph}}\times 100$$

*I_crys_* is the intensity of the ordered peaks, which can be estimated by subtracting the height or area of the disordered background. *I_amorph_* is the intensity of the disordered background.

### 2.8. Space Charge Analysis

Test one conditions:

Pressing conditions: melt pressing at 120 °C for 8 min without pressure, followed by hot pressing at 180 °C with 10 MPa for 8 min, then 15 MPa for 15 min, and finally water-cooling for 5 min to room temperature.

Annealing conditions: vacuum annealing in a drying oven at 70 °C for 12 h, removing crosslinking by-products.

Sample thickness: 200 μm.

Test temperature: 30 °C; applied field strength: 30 kV/mm; pressurization time: 40 min; depolarization time: 10 min.

During the test, the sample temperature was first raised to the set value and stabilized for 30 min to ensure a uniform internal temperature distribution. Then, a DC electric field and pulse electric field were simultaneously applied to the sample. To ensure good contact, an appropriate amount of dimethyl silicone oil was applied between the sample and the electrodes, which also served as an acoustic coupling agent. To prevent surface flashover, an anti-flashover film was added between the sample and the upper electrode.

Test two conditions:

Pressing conditions: preheating at 150 °C without pressure for 5 min, followed by hot pressing at 20 MPa for 20 min, and cooling under pressure at 25 °C for 20 min.

The research team independently assembled the experimental equipment with the following performance parameters: pulse width: 10 ns; pulse frequency: 200 Hz; oscilloscope sampling rate: 2 Gsa; sampling bandwidth: 500 MHz; test field strengths: 10, 20, 30, and 50 kV/mm.

The measurement conditions comply with the “JBT 12927-2016 [15] Test Method for Electric-Acoustic Pulse Space Charge Distribution in Solid Insulating Materials”.

## 3. Results and Discussion

### 3.1. Degree of Branching

The branching structure of polyethylene can be broadly categorized into long branches and short branches. Short branches, such as ethyl and butyl groups, are mainly formed through intramolecular transfer reactions. A substantial presence of randomly distributed short branches can disrupt the regularity of polyethylene molecular chains, potentially leading to difficulties in crystallization or non-crystallinity, further affecting the properties of copolymers, including density, softening point, and hardness. On the other hand, the presence of long branches has a minimal impact on crystalline performance but can influence the flow properties, processing characteristics, and mechanical properties of polymers. The existence of branches results in the irregular arrangement of polyethylene molecules, leading to low crystallinity, poor toughness and tensile strength, as well as a lower melting point. Generally, branching has an adverse effect on the properties of polymeric materials, and the higher the degree of branching and complexity of branches, the greater the impact [16,17,18,19]. Therefore, determining the degree of branching is crucial for characterizing the physical properties of polyethylene and subsequently evaluating the performance of polyethylene products.

**Figure 1:**
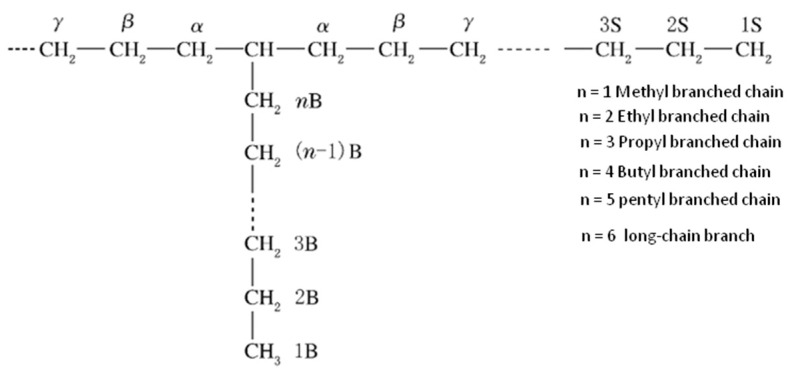
Carbon naming rules for molecular chains of Randall copolymers.

**Figure 2:**
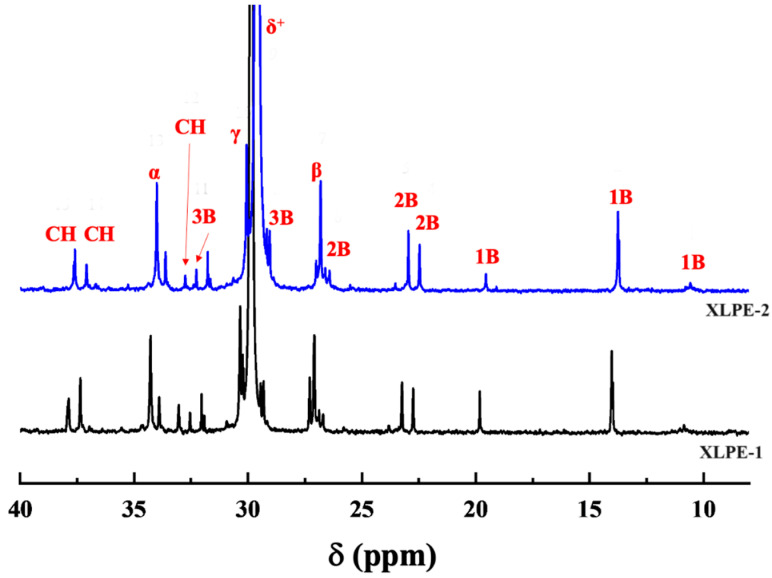
^13^C-NMR spectra of XLPE-1 and XLPE-2 materials.

The chemical shifts of the samples were assigned according to Randall’s method [20] (Figure 1) and the resonance peaks of the individual samples were integrated separately, the degree of branching was calculated, and the results are shown in Figure 2 and Table 2.

From the ^13^C-NMR spectra of the samples (Figure 2), it is evident that the chemical shifts of the two samples are similar. For XLPE-1, the chemical shift at 37.89 represents the resonance peak of the ethyl branching point CH, 37.37 represents the resonance peak of the butyl and hexyl + branching point CH, and 34.28 and 27.10 represent the resonance peaks of the corresponding branching points at the α and β positions. The resonance peak at 33.04 represents the resonance peak of the methyl branching point CH, and 37.09 and 27.10 represent the resonance peaks of the corresponding branching points at the α and β positions. The resonance peak at 29.87 corresponds to the main-chain carbon. The resonance peaks corresponding to the carbon atoms of the four types of branching chains are also listed in Table 2 (see the Supplementary Information). Therefore, both samples exhibit a structure with short branches such as methyl, ethyl, and butyl, as well as long branches with more than six carbons. As indicated in Table 3, for the three types of branching chain structures, the degree of branching for XLPE-2 is consistently lower than that of XLPE-1.

### 3.2. PLM

**Figure 3:**
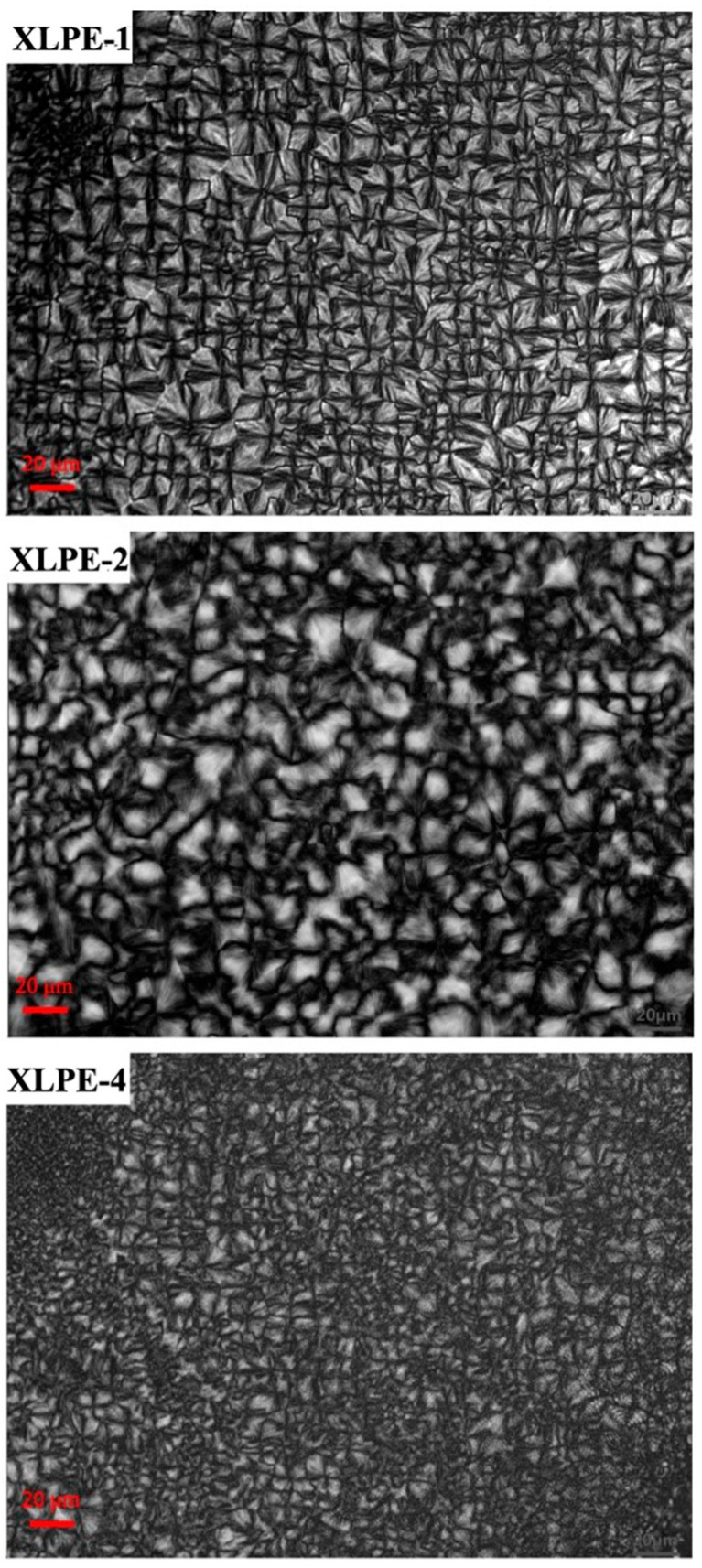
Three polymer materials’ PLM images.

Polarized light microscopy (PLM) is an effective tool for studying the morphology of polymers, providing visual insights into the characteristic structural elements of polymer units [21,22,23,24]. PLM allows for the observation of the shapes of polymer spherulites. The size of spherulites directly influences the mechanical strength of polymers, with larger spherulites leading to a lower impact strength and increased susceptibility to fracture. Spherulite size also affects the transparency of materials; larger spherulites result in a milky and opaque appearance, diminishing transparency. However, if the densities of crystalline and non-crystalline phases are very close, then the material can remain transparent, or if the spherulite size or grain size is smaller than the visible light wavelength, then the material will still appear transparent. The crystalline morphologies of the three samples are depicted in Figure 3. Among them, XLPE-1, XLPE-2, and XLPE-4 all exhibit distinct cross-polarization patterns. However, XLPE-2 displays a more regular grain structure in comparison, presenting predominantly as a fan-shaped radiating pattern. The spherulite size of XLPE-4 is heterogeneous.

### 3.3. XRD

**Figure 4:**
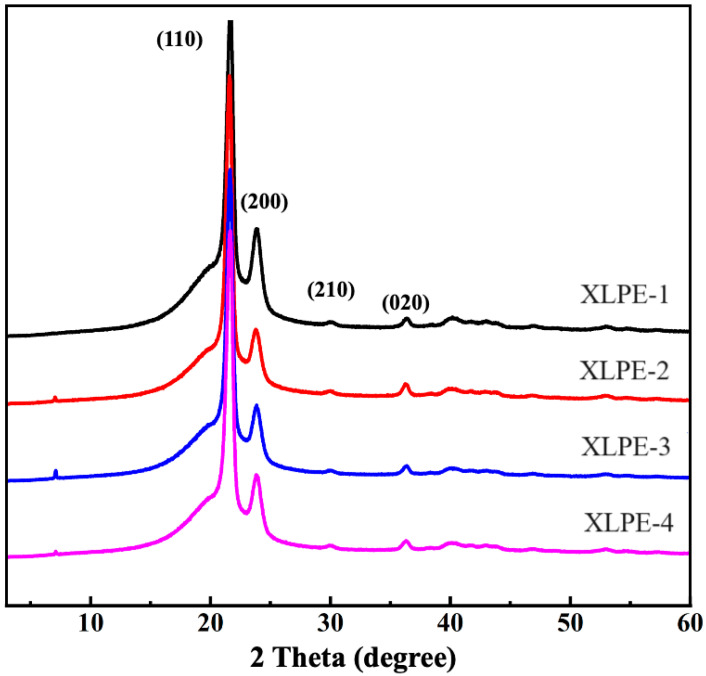
X-ray diffraction spectra of four particle materials.

The primary diffraction angles (2θ) for polyethylene are as follows: 21.6 (110), 23.8 (200), 30.1 (210), and 36.4 (020). Notably, the characteristic diffraction peak intensity for the 210 crystal plane is significantly weaker than that of other crystal planes. From Figure 4, it can be observed that all four samples exhibit both crystalline and semi-crystalline regions, indicating a semi-crystalline nature. Utilizing the Segal equation and integrating the area under the curve, the calculated crystallinity values are presented in Table 4.

### 3.4. Thermal Properties

**Figure 5:**
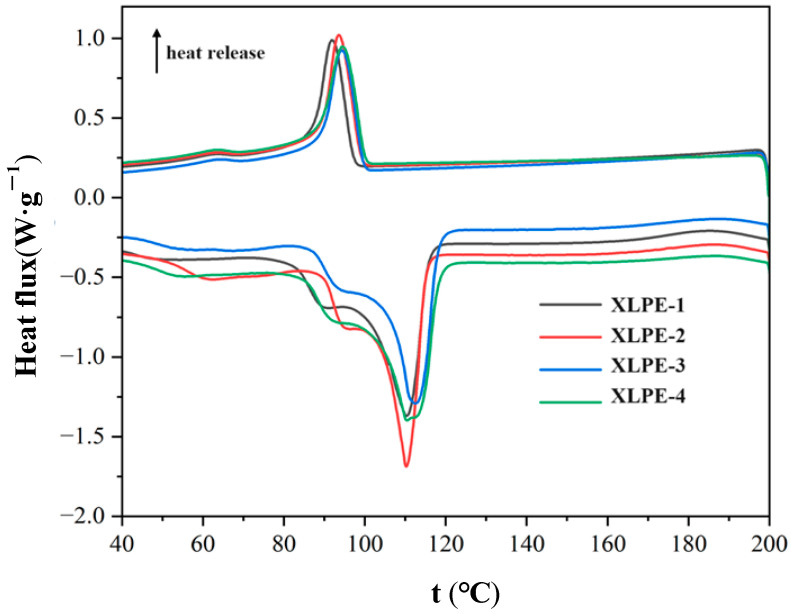
DSC curves of four samples.

From the DSC curves of the samples (Figure 5), we can extract the melt parameters of the materials. Table 5 presents the crystallinity of crosslinked polyethylene calculated using the melting peak. As per the table, the four samples exhibit similar main melting peak temperatures, all around 110 °C. However, XLPE-1 has a higher melting enthalpy, resulting in a higher crystallinity of 34%. Among the four materials, XLPE-4 has the highest melting enthalpy and a crystallinity of 34%, while the crystallinities of XLPE-2 and XLPE-3 are both 30%. The magnitude of crystallinity is influenced by the molecular chain structure of polyethylene, where the presence of long and short branches affects the crystallization process. Additionally, differences in the molecular network structure caused by varying degrees of crosslinking can also impact crystallinity.

### 3.5. Crosslinking Degree Measurement

This parameter reflects the changes in the comprehensive performance of crosslinked polyethylene. Specifically, crosslinking can significantly enhance various properties of polyethylene. For instance, crosslinked polyethylene exhibits a longer service life, improved resistance to aging, better resistance to creep, enhanced resistance to chemical corrosion, improved impact resistance, better wear resistance, superior resistance to environmental stress cracking, and higher temperature resistance. Additionally, crosslinking can enhance the thermal stability and mechanical properties of the material. Therefore, the degree of crosslinking is an important parameter for assessing the performance of crosslinked polyethylene. The degrees of crosslinking, calculated from the values of m_1_~m_3_ in the experimental steps, are presented in Table 6. XLPE-2 has the highest degree of crosslinking, followed by XLPE-1, while XLPE-4 has the lowest degree of crosslinking.

### 3.6. Rheological Behavior

Dynamic rheology is a testing method for oscillatory shear flow under periodic stress or strain conditions. In comparison to static rheology testing, dynamic rheology is typically conducted under small strain conditions, where the testing process minimally influences the material’s own structure, and it primarily reflects the deformability of the melt. The linear viscoelastic response exhibited by polymer materials is highly sensitive to structural changes. By employing dynamic rheology testing methods to measure shear viscosity, dynamic viscosity, storage modulus (G), loss modulus (G″), and other rheological parameters in relation to changes in shear rate or angular frequency, one can analyze these data and relationships with the material’s relative molecular weight, molecular weight distribution (where Mw is the weight-averaged molecular weight and Mn is the number-averaged molecular weight), and branching degree. Therefore, dynamic rheology testing is more effective for studying the morphology and structure of polymer systems. The formation of a crosslinked network structure directly influences the material’s dynamic rheological behavior. The linear viscoelastic response curve of the material can be used to study its crosslinking process. Polymer melts typically exhibit linear viscoelastic behavior in the small strain region, where storage modulus (G) and loss modulus (G″) do not change significantly with strain (γ).

**Figure 6:**
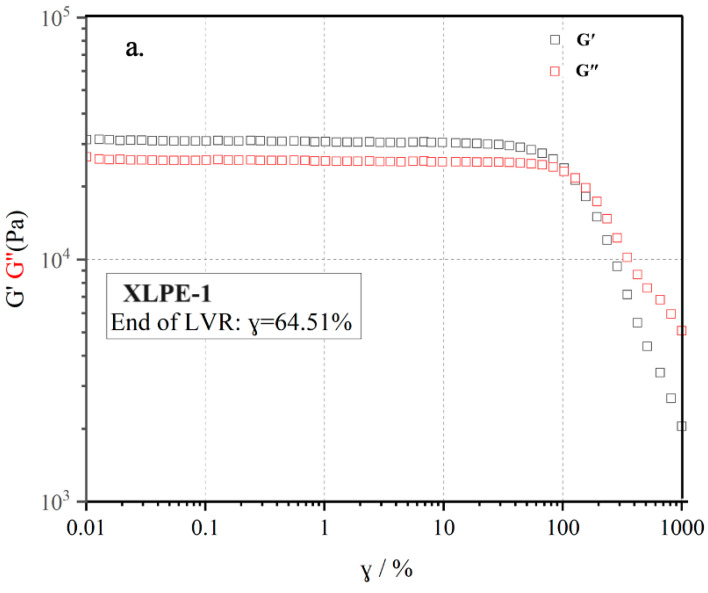

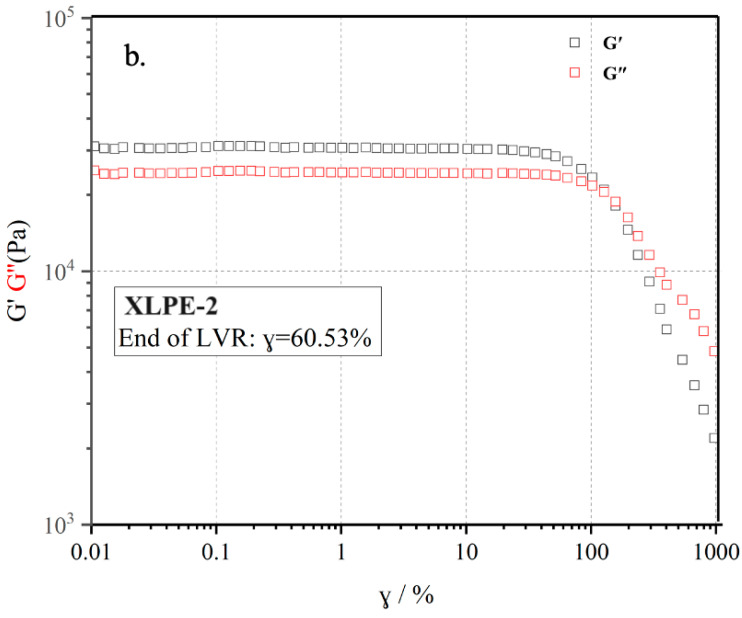
The relationship between the storage modulus G′, loss modulus, and strain γ at 130 °C and ω = 10 rad/s for the two special materials. (**a**) XLPE-1; (**b**) XLPE-2. The gray represents storage modulus G′, the red represents loss modulus G″.

Combined with the previous crosslinking performance, XLPE-1 and XLPE-2 with the best crosslinking performance were selected for rheological property testing. The material’s structure remains undamaged during the testing process. Figure 6 presents the γ sweep at 130 °C for uncrosslinked XLPE-1 and 2. It can be observed that both samples are in the linear region when γ is less than 60%. To ensure that the testing process remained in the linear region, a strain of γ = 1% was chosen.

**Figure 7:**
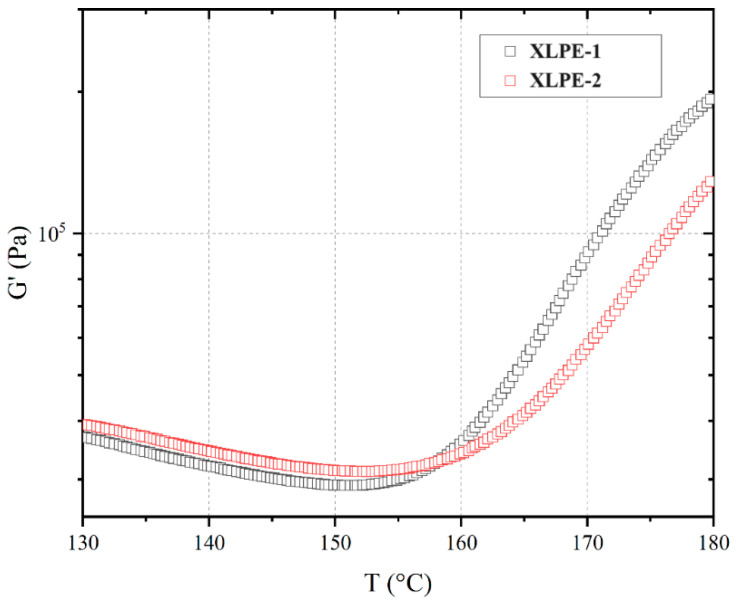
Temperature scan. The relationship between the storage modulus and temperature for XLPE-1 and 2 at a heating rate of 2 °C/min, ω = 10 rad/s, and strain γ = 1%.

During the crosslinking process of polymers, the structure undergoes changes, accompanied by corresponding viscoelastic responses. Figure 7 illustrates the relationship between the storage modulus (G′) and temperature for XLPE-1 and 2 at a heating rate of 2 °C/min. Both samples exhibit a similar temperature dependence trend. Between 130 °C and 155 °C, G′ shows minimal variation with temperature, forming a plateau. When the temperature exceeds 155 °C, G′ sharply increases and reaches an equilibrium trend after 180 °C, with the entire process showing an increase in G′ by an order of magnitude. This indicates that the optimal crosslinking temperature for the system is in the range of 175–180 °C. At lower temperatures, such as 140–160 °C, prolonged heating can also cause crosslinking in the samples. Moreover, at temperatures above 160 °C, the increase in G′ for XLPE-1 is significantly higher than that for XLPE-2, indicating a greater rigidity of the former. Therefore, rheological analysis can be employed to study the viscoelastic response of the system during the crosslinking process at this temperature, providing insights into its structural parameters.

**Figure 8:**
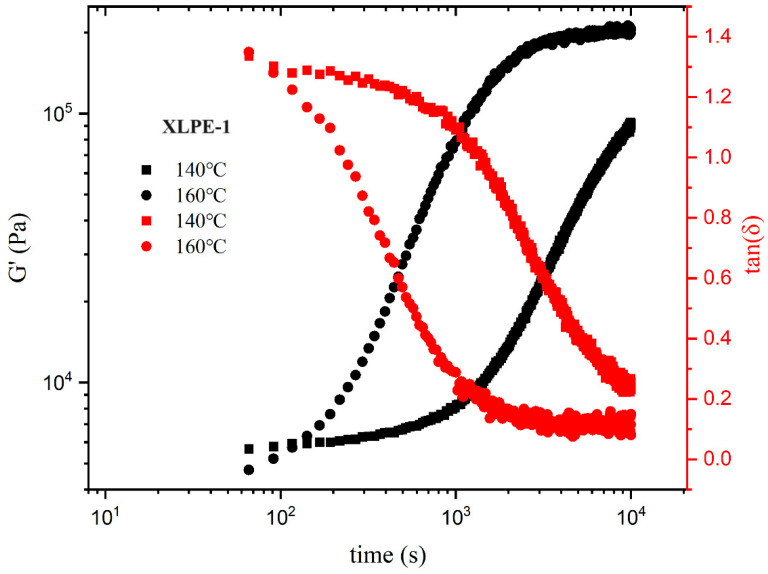
At different temperatures, ω = 0.5 rad/s, and strain γ = 1%, the relationship between the storage modulus G′ and the loss tangent tan δ over time for XLPE-1.

**Figure 9:**
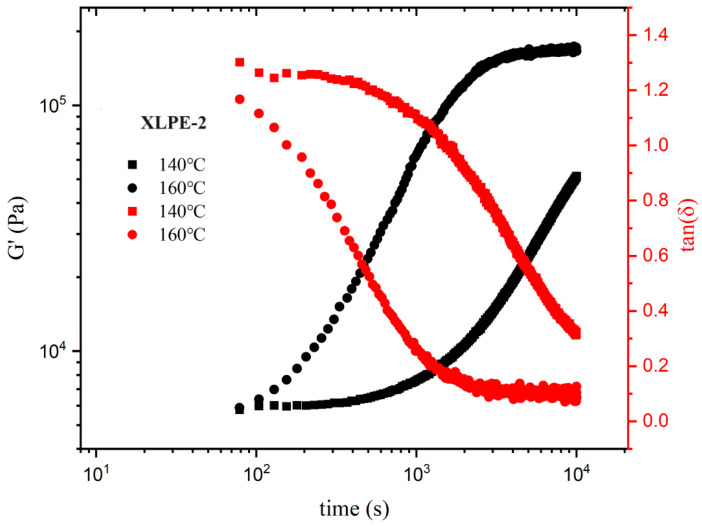
At different temperatures, ω = 0.5 rad/s, and strain γ = 1%, the relationship between the storage modulus G′ and the loss tangent tan δ over time for XLPE-2.

A dynamic time sweep was conducted on two samples at temperatures of 140 °C and 160 °C to investigate the relationship between the storage modulus (G′) and the loss factor (tan δ) over time. Figure 8 and Figure 9 illustrate the time dependencies of G′ and tan δ for XLPE-1 and 2 at different temperatures. It can be observed that, at both temperatures, after a certain period of heating, the system undergoes crosslinking. With increasing time, G′ increases, and tan δ gradually decreases below 1, indicating the gradual improvement of the crosslinked network structure and the enhancement of elastic response. After crosslinking is complete, G′ values reach equilibrium. Notably, the rate of crosslink network formation at 160 °C is significantly higher than at 140 °C, requiring a shorter time for complete crosslinking, resulting in a larger G′ at the same time point.

These results demonstrate a faster crosslinking process at higher temperatures, especially at 160 °C, leading to the formation of a stronger crosslinked network structure. This is crucial for understanding the crosslinking process of the material and for regulating its properties.

Due to its role in characterizing the viscoelastic behavior of polymer materials, the loss factor tan δ is a physical quantity that reflects the energy dissipation. A higher tan δ value indicates greater energy dissipation and poorer material recovery, suggesting slower relaxation of polymer chains under external forces. With an increase in angular frequency, tan δ gradually decreases. This phenomenon is attributed to the accelerated relaxation of polymer chains at higher angular frequencies, leading to the appearance of a plateau with increasing angular frequency. The presence of long branches in the polymer structure can cause a slow decrease in the loss factor in the low-frequency region and an earlier appearance of the plateau [25,26].

**Figure 10:**
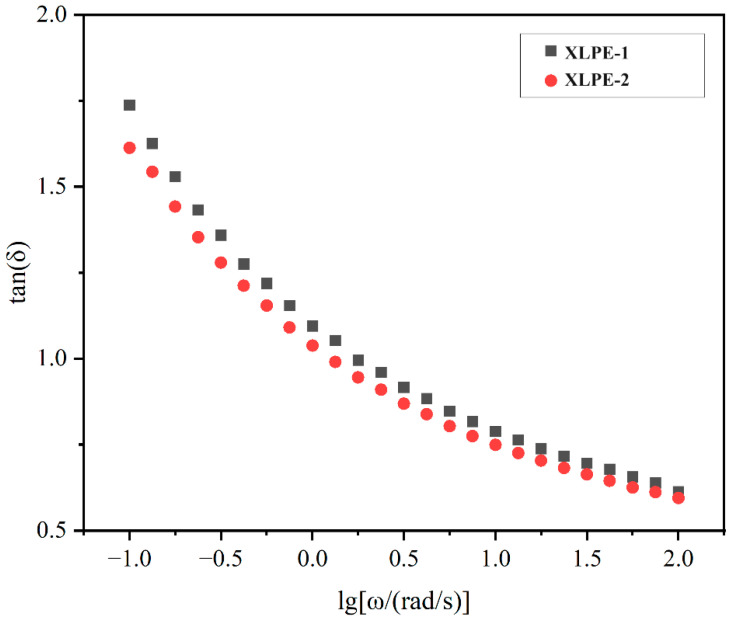
The curve of tan⁡δ variation with frequency.

Figure 10 illustrates a gradual decrease and the appearance of a plateau in the loss factor for both materials, indicating the presence of long branches in their structures. This observation aligns with the results of branching degree analysis conducted using high-temperature liquid nuclear magnetic resonance carbon spectroscopy.

### 3.7. Space Charge Analysis

**Figure 11:**
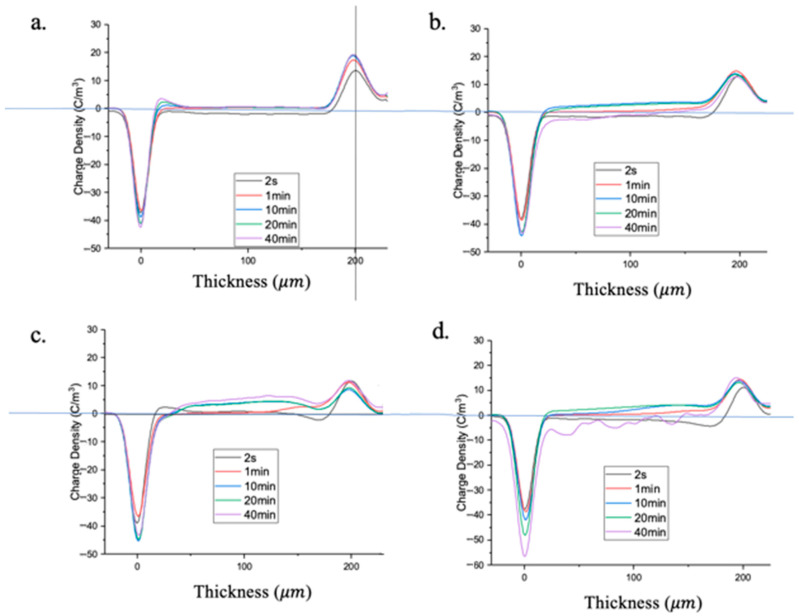
The spatial charge distribution characteristics of four samples with different pressing times under a test field strength of 30 kV/mm. Panels (**a**–**d**) represent the spatial charge distribution of XLPE-1–4 after pressing for 40 min, respectively.

For XLPE-1–4 under test condition 1, the results are shown in Figure 11, depicting the spatial charge distribution under a 30 kV/cm electric field for different pressurization times. The two vertical lines in the figure represent the locations of the cathode (left) and anode (right). All four samples exhibit a significantly higher charge peak at the cathode compared to the anode. This is due to the fact that electron injection at the aluminum electrode interface (the bottom electrode for PEA) is easier than hole injection at the semiconductor electrode interface (the top electrode for PEA), resulting in a higher charge density [27,28].

At 2 s of pressurization, evident polarity charge accumulation is observed near the cathode and anode. For XLPE-1, the spatial charges near the cathode and anode stabilize after 10 min of pressurization. However, for XLPE-2–4, the cathode charge peak increases with pressurization time, and the accumulated opposite-polarity spatial charge near the anode continues to grow. The charge distribution range gradually expands, migrating towards the cathode direction.

For XLPE-4, positive charges mainly accumulate near the electrode, representing same-polarity charge accumulation. In XLPE-2 and 3, the internal positive charges gradually increase, originating from impurity dissociation, indicating opposite-polarity charges.

With increasing pressurization time, the accumulation of same-polarity spatial charges inside the samples gradually increases, penetrating the entire sample. The waveform of the spatial charge obtained by PEA represents net charge quantity, and the accumulation of same-polarity spatial charges can mask the accumulation of opposite-polarity charges. For positive spatial charges, they mainly arise from impurity dissociation, leading to a more severe impurity dissociation inside the sample. XLPE-1 does not exhibit obvious spatial charge accumulation, indicating good spatial charge suppression effects. Compared to XLPE-2, XLPE-3 shows a slight accumulation of opposite-polarity spatial charges near the cathode. Therefore, among XLPE-1–4, XLPE-2 and 3 demonstrate better performance, with XLPE-2 exhibiting the best performance.

Under test condition two, for all four samples, electric fields of 10, 20, 30, and 50 kV/cm were applied, and the results are shown in Figures S2–S5. It can be observed that for XLPE-2, 3 and 4, when the electric field is increased from 10 kV/cm to 50 kV/cm, significant spatial charge injection occurs, especially for XLPE-2 and 4, where sustained transport of spatial charge packets is observed, which is highly unfavorable for reducing spatial charge accumulation in the dielectric. XLPE-1 is expected to have better performance.

Combining the results obtained under these two different test conditions, XLPE-1 exhibits better performance.

## 4. Conclusions

(1) According to the ^13^C-NMR results, XLPE-1 and XLPE-2 both show the existence of short-chain branch chains and long-chain branch chains, which is consistent with the rheological test results. In addition, the degree of branching of XLPE-2 is lower than that of XLPE-1. Through DSC analysis, the crystallinity of XLPE-2 is lower than that of XLPE-1, and in general, the introduction of branches will reduce the crystallinity. For the above abnormal phenomenon, by testing the crosslinking degree of XLPE-2 and XLPE-1, it is concluded that the crosslinking degree of XLPE-2 is higher than that of XLPE-1, indicating that XLPE-2 with fewer branches has more effective crosslinking under the action of crosslinking agents, forming a three-dimensional network structure and reducing the crystallinity of the polymer.

(2) With the increase in DCP content, the crosslinking degree of four kinds of crosslinked samples first increased and then decreased. Combined with their space charge analysis, XLPE-2, XLPE-3, and XLPE-4 showed polar-like space charge aggregation, and XLPE-1 showed better inhibition of the space charge effect. For XLPE-3 and XLPE-4, the internal charge increase is considered to be caused by excessive DCP decomposition and impurity dissociation. Compared to XLPE-3, the XLPE-4 relay process is more severe, which is consistent with its highest DCP content. For XLPE-2, although a high crosslinking degree will enhance the mechanical stability of the polymer, due to the high crosslinking degree, polar charges accumulate near the cathode and anode.

## Figures and Tables

**Table 1:** Composition of sample.

	LDPE/g	DCP/g	Antioxidant 300/g	AMSD/g
XLPE-1	100	0.5	2	1
XLPE-2	100	1.0	2	1
XLPE-3	100	1.5	2	1
XLPE-4	100	2.0	2	1

**Table 2:** Chemical shifts in the ^13^C-NMR spectrum of XLPE-1.

Branching Chain	Main Chain Carbon Atoms (ppm)			Branching Carbon Atoms (ppm)		
	Branching Point	α Position	β Position	1B	2B	3B
Methyl	33.04	37.09	27.10	19.82	-	-
Ethyl	37.89	34.28	27.10	10.85	26.88	-
Butyl	37.37	34.28	27.10	14.03	23.24	29.32
Hexyl+	37.37	34.28	27.10	14.03	22.75	32.55

**Table 3:** Branching degrees of XLPE-1 and XLPE-2.

Branching Chain	Branching Degree/1000C	
	XLPE-1	XLPE-2
Methyl	5.2	1.4
Ethyl	8.5	7.5
Butyl and Hexyl+	9.6	3.7

**Table 4:** Crystallinity of four particle materials obtained by XRD method.

Sample	Crystallinity %
XLPE-1	24.6
XLPE-2	26.5
XLPE-3	24.1
XLPE-4	25.1

**Table 5:** Crystallinity calculation for different granules.

Sample	Melt Parameters		Crystallinity $\chi_{c}$%
	$T_{m}$ (°C)	$H_{f}$ ($J\cdot g^{-1}$)	
XLPE-1	110.24	98.85	34
XLPE-2	109.84	88.18	30
XLPE-3	112.13	88.62	30
XLPE-4	110.04	98.24	34

Note: $T_{m}$ represents the peak value of the absorption peak; $H_{f}$ represents the area of the absorption peak.

**Table 6:** The crosslinking degrees of the four granular materials.

Sample	Degree of Crosslinking %
XLPE-1	80.2 ± 1.1
XLPE-2	82.2 ± 1.4
XLPE-3	79.4 ± 1.1
XLPE-4	77.7 ± 1.4

## Data Availability

Data are contained within the article.

## Supplementary Materials

The following supporting information can be downloaded at: https://www.mdpi.com/article/10.3390/polym16050676/s1. Figure S1. NMR 13C spectra of XLPE-1 and XLPE-2. Figure S2. The spatial charge distribution under test condition two for the four samples with a testing electric field of 10 kV/mm is illustrated in panels a–d for samples 1–4, respectively, after applying pressure for 60 min. (a, XLPE-1; b, XLPE-2; c, XLPE-3; d, XLPE-4). Figure S3. The spatial charge distribution under test condition two for the four samples with a testing electric field of 20 kV/mm is illustrated in panels a-d for samples 1–4, respectively, after applying pressure for 60 min. (a, XLPE-1; b, XLPE-2; c, XLPE-3; d, XLPE-4). Figure S4. The spatial charge distribution under test condition two for the four samples with a testing electric field of 30 kV/mm is illustrated in panels a-d for samples 1–4, respectively, after applying pressure for 60 min. (a, XLPE-1; b, XLPE-2; c, XLPE-3; d, XLPE-4). Figure S5. The spatial charge distribution under test condition two for the four samples with a testing electric field of 50 kV/mm is illustrated in panels a-d for samples 1–4, respectively, after applying pressure for 60 min. (a, XLPE-1; b, XLPE-2; c, XLPE-3; d, XLPE-4).
