# Supplementary material for: Influence of Crosslink Density on Electrical Performance and Rheological Properties of Crosslinked Polyethylene
Source: Polymers (Basel). 2024 Mar 1;16(5):676. doi: 10.3390/polym16050676 (PMC10934902; doi:10.3390/polym16050676)
Supplement: Supplementary file 1 [file polymers-16-00676-s001.zip › polymers-2830564-supplementary.pdf]

Supporting Information

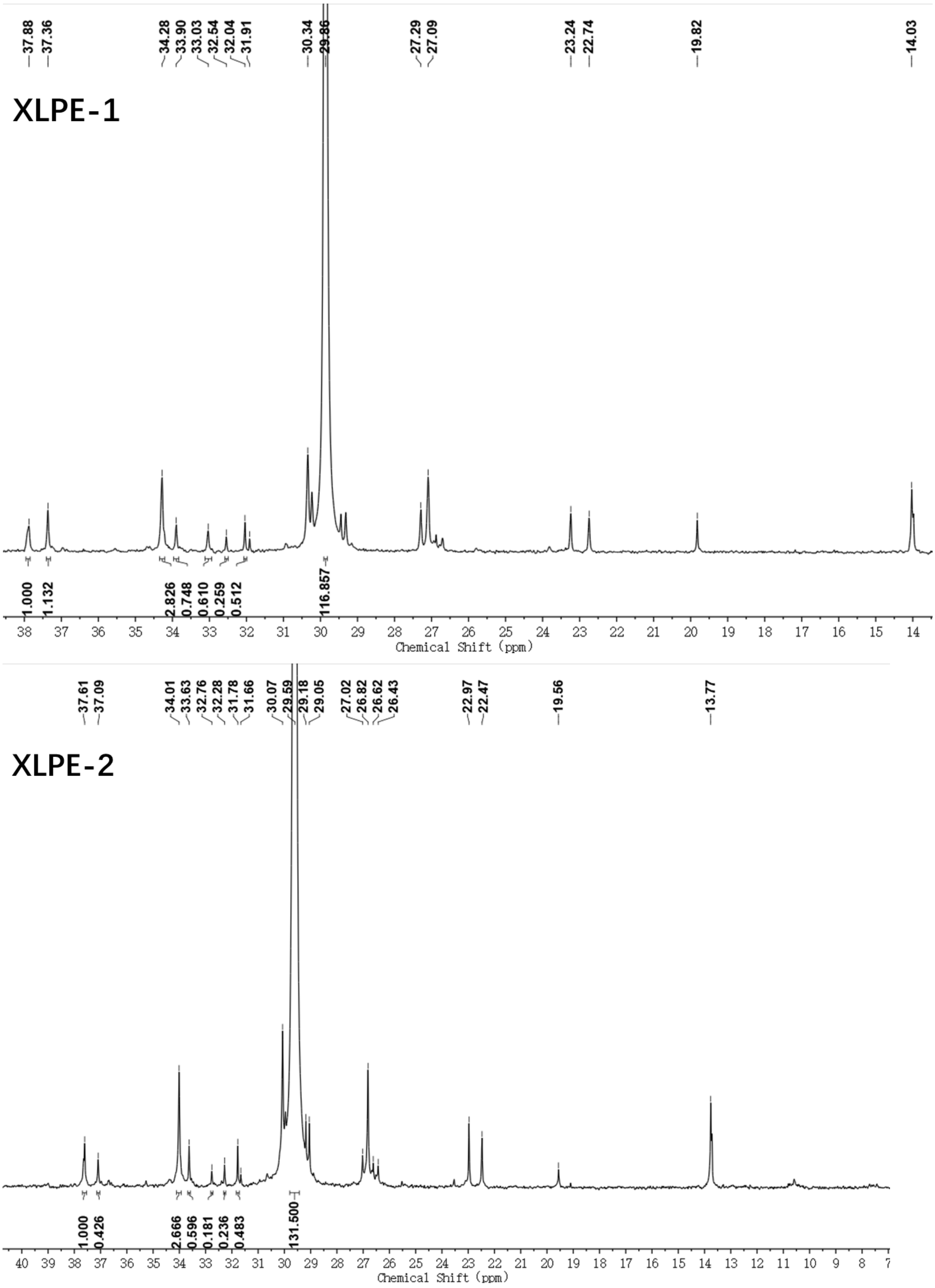

Figure S1. NMR ^{13}C spectra of XLPE-1 and XLPE-2

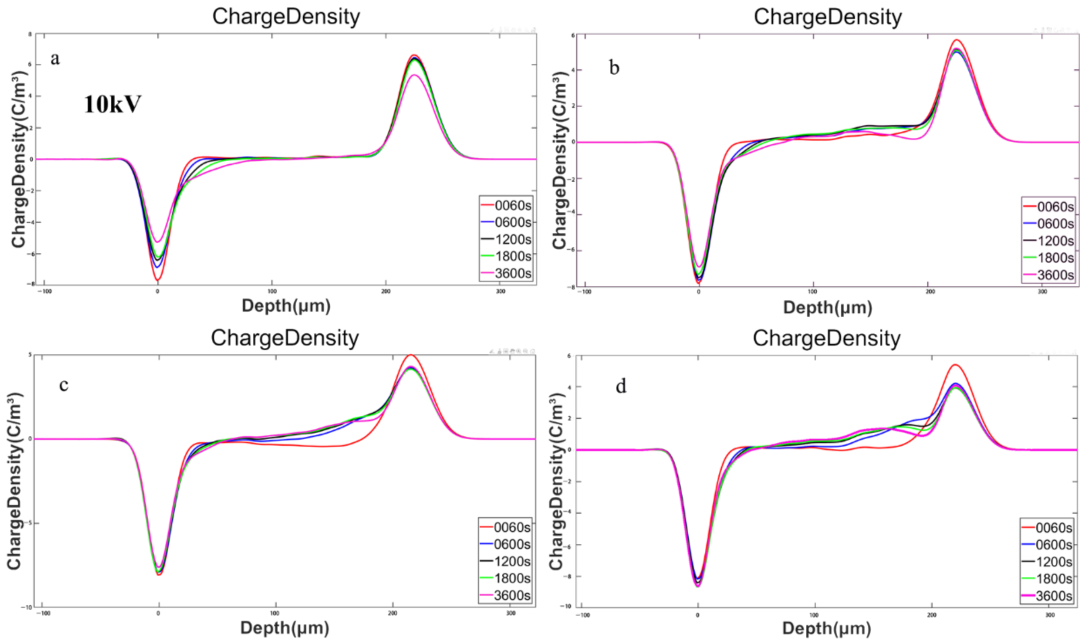

Figure S2. The spatial charge distribution under test condition two for the four samples with a testing electric field of 10 kV/mm is illustrated in panels a-d for samples 1-4, respectively, after applying pressure for 60 minutes. (a,XLPE-1;b,XLPE-2; c, XLPE-3; d, XLPE-4)…

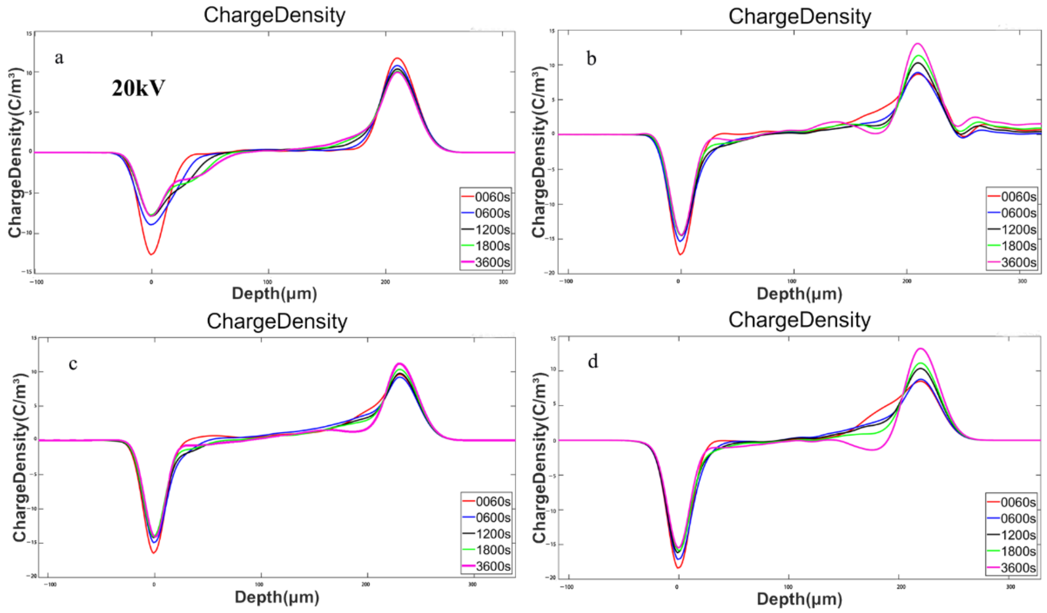

Figure S3. The spatial charge distribution under test condition two for the four samples with a testing electric field of 20 kV/mm is illustrated in panels a-d for samples 1-4, respectively, after applying pressure for 60 minutes. (a,XLPE-1;b,XLPE-2; c, XLPE-3; d, XLPE-4)

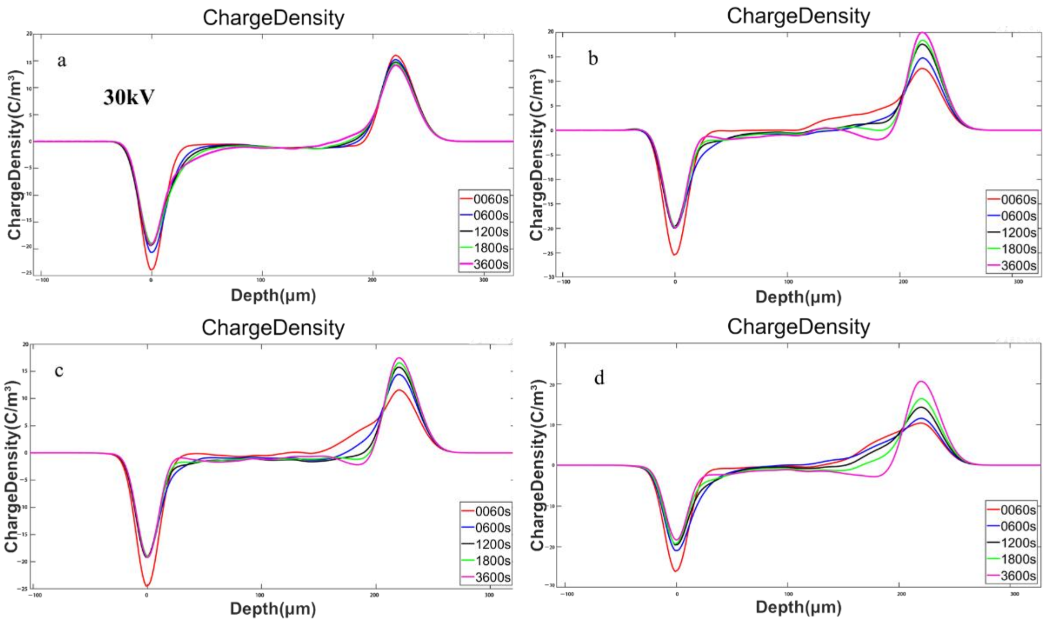

Figure S4. The spatial charge distribution under test condition two for the four samples with a testing electric field of 30 kV/mm is illustrated in panels a-d for samples 1-4, respectively, after applying pressure for 60 minutes. (a,XLPE-1;b,XLPE-2; c, XLPE-3; d, XLPE-4)

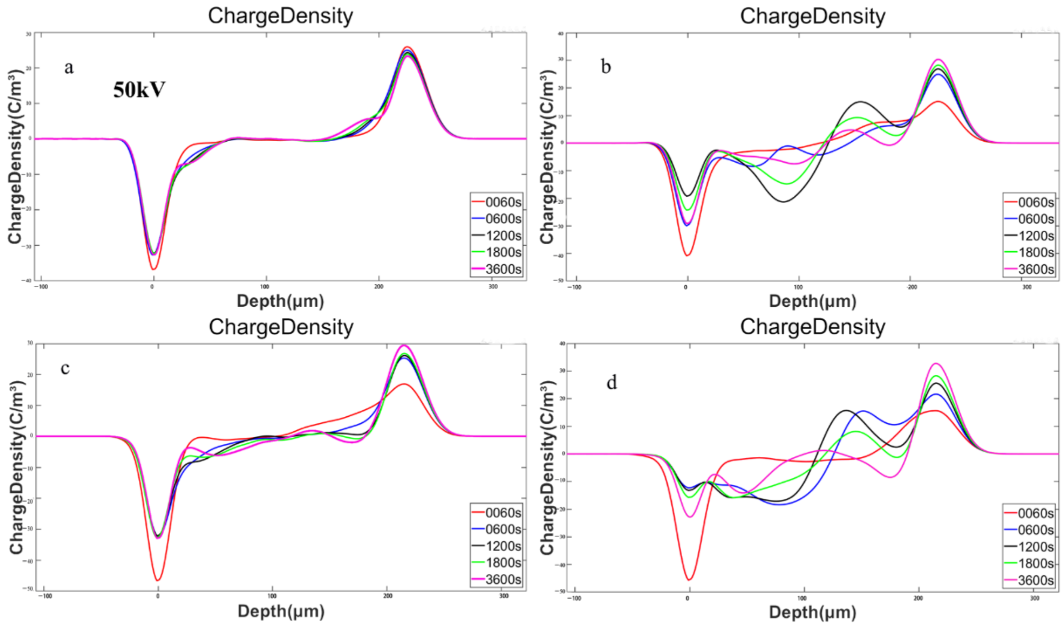

Figure S5. The spatial charge distribution under test condition two for the four samples with a testing electric field of 50 kV/mm is illustrated in panels a-d for samples 1-4, respectively, after applying pressure for 60 minutes. (a,XLPE-1;b,XLPE-2; c, XLPE-3; d, XLPE-4)
